# Supplementary material for: Perturbation of the titin/MURF1 signaling complex is associated with hypertrophic cardiomyopathy in a fish model and in human patients
Source: Dis Model Mech. 2019 Nov 15;12(11):dmm041103. doi: 10.1242/dmm.041103 (PMC6899042; doi:10.1242/dmm.041103)
Supplement: Supplementary information [file dmm-12-041103-s1.pdf]

Figure S1. Normal vascular formation was observed in the *nsh* mutant.

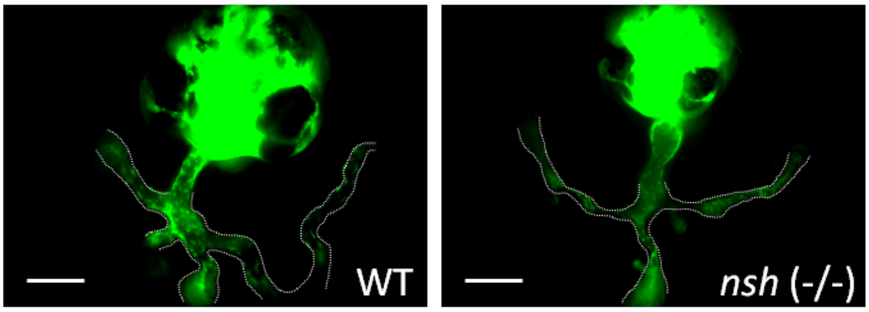

Crossing with *fli1*-GFP transgenic medaka revealed normal vascular endothelial cells and normal blood vessel formation in the WT and *nsh* mutant at 3 dpf. Scale bars, 200 µm.

Figure S2. Measurement of heart weight/body weight ratios

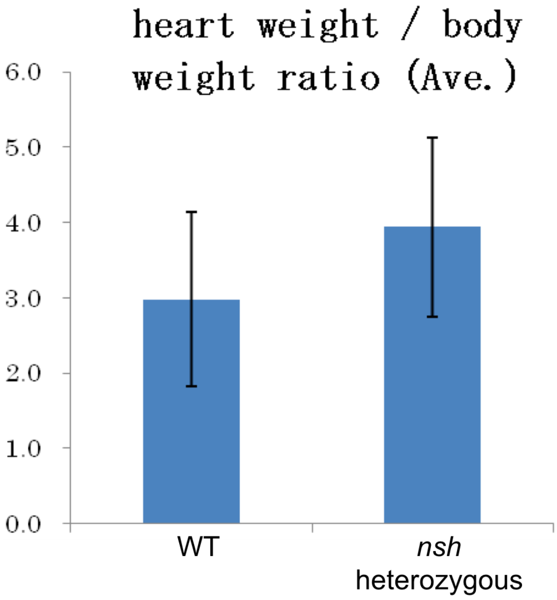

The ratio of the heart weight to body weight. Values are mean +/- SEM.

Disease Models & Mechanisms • Supplementary information

Figure S3. Sarcomeric structures were disrupted in the *nsh* skeletal muscles.

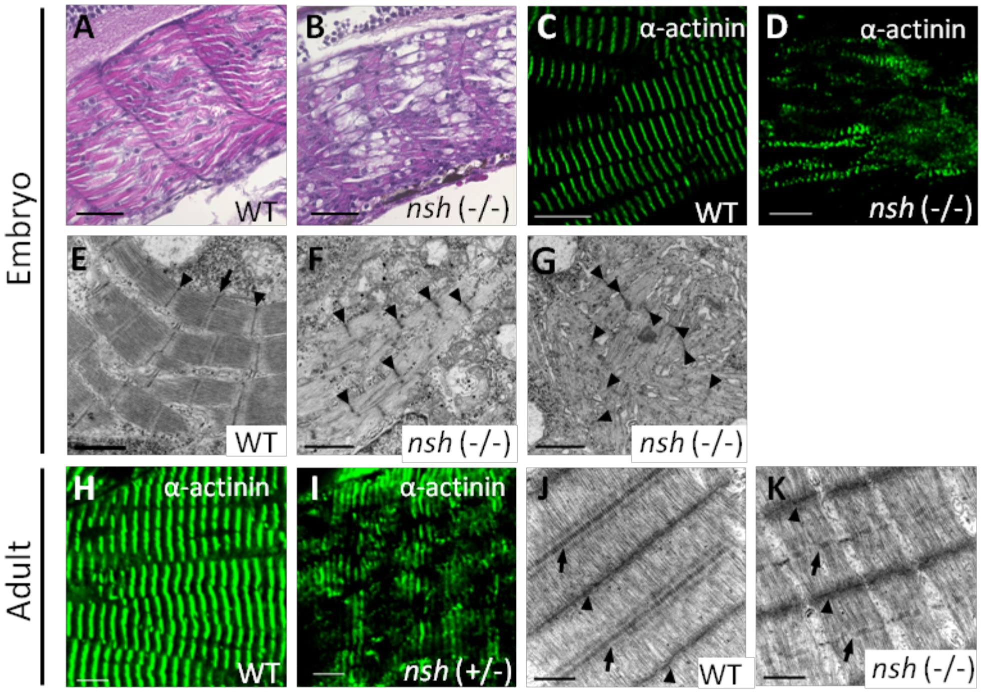

A and B, Longitudinal sections of medaka embryonic skeletal muscle at 5 dpf, stained with hematoxylin/eosin. WT muscle showed regularly aligned myofibers, whereas the *nsh* myofibers were disorganized. C and D, Embryonic skeletal muscle of WT and *nsh* mutant (*nsh* -/-) at 3 dpf stained for α-actinin. Sarcomeric structures were disrupted in the *nsh* mutant (D). E through G, Transmission electron microscopy of medaka embryonic skeletal muscles at 5 dpf. (E) In the WT skeletal muscles, myofibrils were assembled regularly. F and G, In the *nsh* mutant, skeletal muscles showed severely disturbed sarcomeric integrity with ruptured myofibrils, blurry Z-discs and M-lines, irregular sarcomere length, lack of thick filaments, and pronounced myocyte disarray (G). H and I, Adult skeletal muscle from WT and *nsh* heterozygotes (*nsh* +/-) stained for α-actinin. Patchy staining was observed occasionally in the *nsh* heterozygotes (I). J and K, Transmission electron microscopy of medaka adult skeletal muscles. J, WT demonstrated well-defined Z-discs and M-lines in skeletal myofibrils. K, Skeletal myofibrils were visible in the *nsh* heterozygotes. Although the mutant displayed well-formed Z-discs and mature-appearing myofibrils, it also demonstrated occasionally disrupted M-lines. Z-discs (arrowhead) and M-lines (arrow) are indicated. Scale bars, 30 µm in (A and B), 10 µm in (C and D), 1 µm in (E through G), 5 µm in (H and I), and 0.5 µm in (J and K).

Disease Models & Mechanisms • Supplementary information

Figure S4.

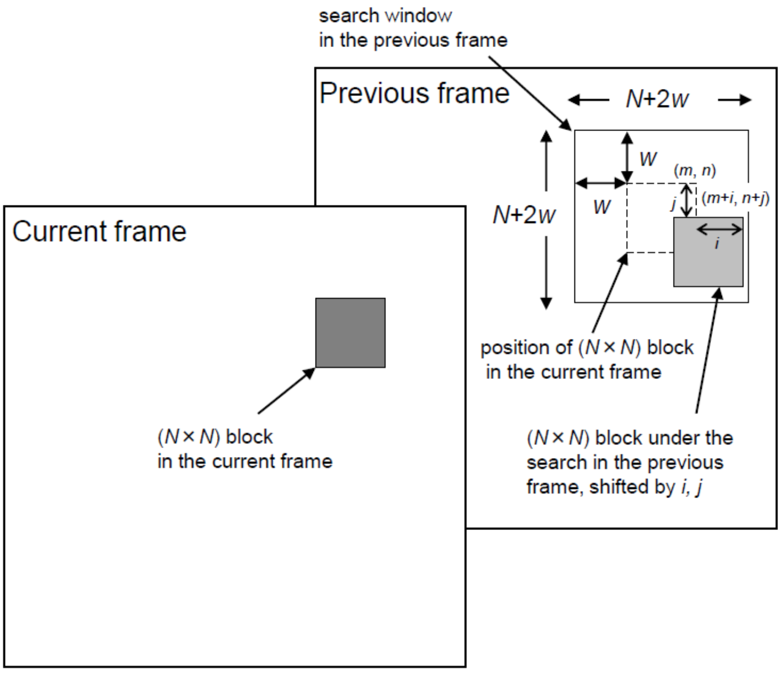

The Schematics of sequential frames in a search window. Each frame was divided into square blocks of $N \times N$ pixels. For a maximum motion displacement of w pixels per frame, the current block of pixels was matched to the corresponding block at the same coordinates in the previous frame within a square window of width $N + 2w$. In this study, we set the parameters, N= 16 and W= 4.

Table S1. Primers used for PCR amplification and sequencing of human *TTN*.

**Exon**	Forward primer (5'>3')	Reverse primer (5'>3')
**296**	ctatgttgattatatttccccaag	ggtaaattgttacgaattctgaatg
**297**	cattcagaattcgtaacaatttacc	aattgggtgactgaacatcaac
**298**	gttgatgttcagtcacccaatt	gttaacattcagaatcagaggtg
**299**	ggaaaacacaaatatatgagtcattg	cccacatataaattgtaactcaatc
**300**	gaatttgttaggtggcacagc	gtctatggcaattattgcagaaag
**301**	ctttctgcaataattgccatagac	caagtattataagccaatgactttc
**302**	gaaagtcattggcttataatacttg	ggaccaaacatggcttgcttc
**303**	gaagcaagccatgtttggtcc	ggaattagccttaacttgtttag
**304**	tatacaaggacaaaggattacag	caattgtggtggttttcatagtg
**305**	cactatgaaaaccaccacaattg	tctgaatctttgctatttcctttc
**306**	gctttaataaagctgcctgtttc	caagttaataactcatttagcctc

Disease Models & Mechanisms • Supplementary information

**307a**	gaggctaaatgagttattaacttg	cctacacggagccatctttc
**307b**	tctgtcaacttaacatggactg	aggtgtaagatgtttctatgcc
**307c**	agtcaaagggactgatcagg	ctgttggttagtttcagccagg
**307d**	agccacagacatgtggtcac	gtaccaagtcacttgggtag

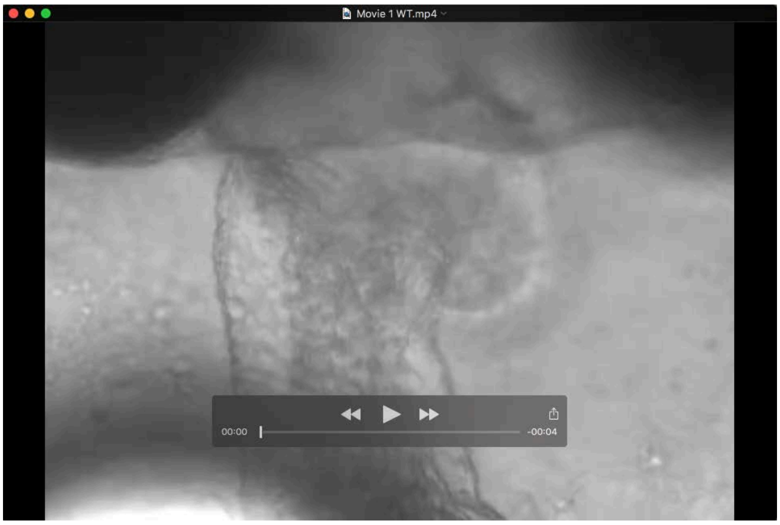

Movie 1. Contracting heart of a WT embryo.

Representative frontal view of a WT heart at 5 dpf. Atrium was on this side and ventricle was deeper side. Blood flow was observed in the myocardial wall.

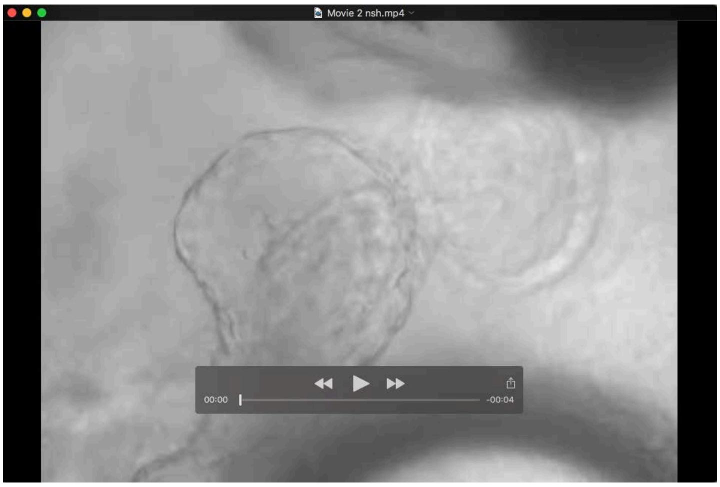

Movie 2. Contracting heart of a *nsh* mutant embryo.

Representative frontal view of a mutant heart at 5 dpf. The *nsh* mutant heart had lost elasticity, beat in a rigid manner and showed increased thickening of the myocardial wall, even without blood flow.
